# Rethinking Obesity Counseling: Having the French Fry Discussion

**DOI:** 10.1155/2014/525021

**Published:** 2014-10-19

**Authors:** Jonathan Bonnet, Aaron George, Pippa Evans, Mina Silberberg, Diana Dolinsky

**Affiliations:** ^1^Department of Community and Family Medicine, Duke University Health System, P.O. Box 104425, Durham, NC 27710, USA; ^2^Duke Primary Care, Duke University Health System, 1820 Hillandale Road Suite 24B, Durham, NC 27705, USA; ^3^113 Trail One, Burlington, NC 27715, USA

## Abstract

Childhood obesity is a complex problem that warrants early intervention. General recommendations for obesity prevention and nutrition counseling exist. However, these are notably imprecise with regard to early and targeted interventions to prevent and treat obesity in pediatric populations. This study examines family medicine primary care providers' (PCPs) perceived barriers for preventing and treating pediatric obesity and their related practice behavior during well-child visits. *Methods.* A written survey addressing perceived barriers and current practices addressing obesity at well-child visits were administered to PCPs at eleven family medicine clinics in the Duke University Health System. *Results.* The most common perceived barriers identified by PCPs to prevention or treatment of obesity in children were families not getting enough exercise (93%) and families too often having fast food meals (86%). Most PCPs do not discuss fast foods at or prior to the twelve-month well-child visit. The two-year visit is the first well-child visit at which a majority of PCPs (68%) discuss fast food. * Conclusion.* No clear consensus exists as to when PCPs should discuss fast food in early well-child checks. Previous research has shown a profound shift in children's dietary habits toward fast foods, such as French fries, that occurs between the one- and two-year well-child checks. Consideration should be given to having a “French Fry Discussion” at every twelve-month well-child care visit.

## 1. Introduction

Obesity is a major public health problem with over two-thirds of Americans overweight and greater than one-third obese [1]. While new data have suggested a leveling off of the prevalence of childhood obesity, 12.4% of kindergarteners are obese and overweight five-year olds are four times as likely as normal weight five-year olds to become obese [2, 3]. It has been well documented that obesity leads to cardiac, metabolic, and other systemic health derangements [4]. It has also been shown that these unfavorable changes may start as early as three years of age [5]. This emphasizes the importance of early intervention and prevention.

Well-child checks have long been recognized as opportunities to foster healthy growth and development. These regular visits, which are performed with a health care provider, traditionally occur during the newborn period, at one, two, four, six, nine, twelve, fifteen, eighteen, and twenty-four months and every year thereafter. The goal is to provide continuity of care and allow for anticipatory guidance to be given. Recognizing that diet and nutrition are linked with obesity, it is important to address these topics. However, guidelines on nutrition counseling remain vague, and visit time is constrained by a multitude of other encouraged and essential components. Yet studies show that when it comes to counseling during well-child checks, less is more and focusing on fewer topics is more effective [6].

General recommendations exist for age specific pediatric guidelines for obesity prevention and nutrition counseling. However, these are notably imprecise with regard to early and targeted interventions to prevent and treat obesity in pediatric populations.

Regarding nutrition counseling during well-child care within the first year of life, the American Academy of Pediatrics (AAP) Bright Future Guidelines contains goals for temporal introduction of foods rather than composition of diet. Even at the one-year visit, recommendations are limited to providing children with “nutritious food and healthy snacks.” Surprisingly, there is no mention of nutrition guidance at the fifteen- or eighteen-month well-child checks and it is not until the two-year visit that the subject of obesity is first addressed in the guidelines [7]. Meanwhile, research shows that a rapid transition occurs between the one- and two-year well-child checks, when diet habits seemingly shift to favor fast foods, with the French fry as the number one consumed vegetable consumed by two-year olds [8].

The World Health Organization (WHO) strongly advocates for exclusive breastfeeding in the first six months of life and offers recommendations on complimentary feeding habits. These include continuing on-demand breastfeeding until two years of age and starting at 6 months of age, gradually increasing the number of feedings, consistency, and variety of other foods. For a nonbreastfed child, WHO further recommends providing four to five meals per day, with one or two healthy snacks aimed to meet the child's nutritional needs [9]. These WHO guidelines are appropriately aimed toward a global audience, with an emphasis on preventing malnutrition and ensuring adequate growth.

Current literature on prevention and treatment for obesity has overwhelmingly focused on adolescent and school aged populations. Meanwhile, diet and lifestyle habits begin to be ingrained much earlier. The AAP recommends screening for obesity starting at two years, while United States Preventive Services Task Force (USPSTF) suggests waiting until age six [7, 10]. These recommendations bypass a likely window of opportunity for primary prevention prior to the second year of life, and—in the case of the USPSTF—prior to six years.

Given these vague universal recommendations, we hypothesize that significant variability exists in clinical practice. It has been demonstrated that there is variability among pediatric primary care practices regarding obesity counseling, attitudes, and perceptions [11]. Some prior studies have focused on family medicine primary care providers' (PCPs) beliefs and practices pertaining to childhood obesity [12]. However, many of these studies have placed emphasis more specifically on evaluation of treatment modalities, perception of obesity as a disease, and physician training [13–18]. Further, much of the previous work on childhood obesity appears directed beyond the age of five years, with few interventions studied between two and six years of age [19]. A paucity of literature has focused on intervention prior to two years of age. This study builds upon the existing literature by offering a family medicine perspective and focusing specifically on perceived barriers and anticipatory guidance discussed at the early well-child checks—particularly those prior to the second year of life. This is further warranted as much of the current literature occurred prior to the updated 2008 AAP Bright Futures Guidelines [7, 20]. Given the recent emphasis on primary care and the likely influx of pediatric patients via the Accountable Care Act, family physicians will continue to provide a substantial amount of care for pediatric populations.

The goal of the present study is to assess the perception of family medicine PCPs in a university based family medicine network surrounding the barriers of preventing and treating obesity in the young child and to analyze PCPs reported behaviors at well-child checks. In addition, the present study allows for a comparison of attitudes and practice between family medicine PCPs and pediatric PCPs who participated in an earlier study using a similar survey [11].

## 2. Materials and Methods

### 2.1. Participants

The sample included all family medicine physicians, physician assistants (PAs), and nurse practitioners (NPs) at eleven family medicine Duke Primary Care sites in Duke University Health Systems.

### 2.2. Survey Design

The survey used in the present study was developed based on a similar study performed in pediatric practices, current epidemiological literature, and recommendations from the American Academy of Pediatrics (AAP) and the United States Preventive Services Task Force (USPSTF) [6, 10, 11]. Previous authors were contacted for permission to utilize a similar survey tool. The survey contained three independent sections, each with a brief introduction. The sections were as follows: (i) perceived barriers in treating obesity, (ii) current PCP practices at well-child checks, and (iii) demographic information.

### 2.3. Procedures

Eleven Duke Primary Care practices were asked and agreed to participate. Introductory phone calls with follow-up emails were sent in January 2012, prior to the in-person survey administration. An agreed upon morning or lunch hour time was arranged to include a ten- minute presentation explaining the goals of the project, with subsequent survey administration to all PCPs who attended the meetings. Written surveys were completed by the respondents anonymously and collected on site. Participation was completely voluntary and there were no financial incentives. An agreement was made to present the study findings to the participating sites at the conclusion of the study. Data collection was performed during January and February 2012 and the data were analyzed in March 2012.

### 2.4. Data Analytic Plan

Demographic data are presented as percentages/proportions. For questions related to perceived barriers, we calculated the percentage of respondents who indicated how important each issue was on a Likert scale 1–5 (Not Important, Slightly Important, Moderately Important, Very Important, and Critically Important), as well as the combined percentage of those who responded either Very Important (4) or Critically Important (5). For PCP behaviors assessing anticipatory guidance, multiple responses were accepted (i.e., mark all well-child checks that apply). These data are presented as percentages/proportions.

### 2.5. Institutional Review Board

This study was reviewed by the Research Advisory Board of the Primary Care Research Committee and the Institutional Review Board of Duke University and was found to be an exempt study (PRO00034169).

## 3. Results

### 3.1. Respondents

Surveys were completed by 56 of the 78 family medicine PCPs (41 family medicine physicians, 8 physician assistants, and 7 nurse practitioners) at the 11 participating clinics, for a 72% response rate. 55% of the respondents were female. Approximately 1/3 (35%) were 40–49 years old, 1/3 (33%) were 30–39, 27% were 50+ years old, and 5% were twenty through twenty-nine years old. The majority of PCPs were medical doctors or doctors of osteopathic medicine (MD/DO) (73%), with physician assistants and nurse practitioners making up 14% and 13%, respectively. The average reported body mass index (BMI) of respondents was 24.71 kg/m^2^ (7 of the 54 respondents did not complete the BMI measures). Approximately 1/3 (33%) of the PCPs were classified as overweight and 0.6% were in the obese category. Reported BMI closely mirrored PCPs perception of their weight, as 30% indicated they were overweight and 2% believed they were obese.

### 3.2. Perceived Barriers to Treating Pediatric Obesity

PCPs were asked to rate the relative importance of specific barriers to preventing or treating overweight or obese children. These questions related to children of all ages and focused on many factors including the child, parents, family unit, influence of society, and PCP factors. The five barriers that were most often rated as either Very Important or Critically Important were as follows: (i) families do not get enough exercise (93%); (ii) families often have fast food meals (86%); (iii) parent is not motivated to change diet or lifestyle (81%); (iv) families watch too much TV (79%); and (v) child is not motivated to change diet or lifestyle (75%) (Table 1). The following barriers were rated as Very Important or Critically Important by 60–72% of PCPs: parent is unaware that child is overweight, parents are overweight so they are not concerned that child is overweight, families are too busy to eat home cooked meals, healthy foods are too expensive, TV advertisements promote unhealthy foods, PCPs have limited time to discuss nutrition, and PCPs are frustrated with the low success rate of treating overweight children. Barriers that PCPs were less likely to rate as Very Important or Critically Important were as follows: overweight child does not act sick, overweight child is a “good eater,” parents do not have time to shop for healthier foods, families are too busy to eat meals together, healthy lifestyle habits are too complicated to follow, published reports about diet and nutrition are often confusing, school lunches promote unhealthy eating habits, PCPs' time constraints, lack of training to treat overweight children, compensation for obesity treatment, access to nutritionists, and PCPs' weight status or body mass index (Table 1).

### 3.3. Current Practices: Occurrence of Anticipatory Guidance

PCPs were asked to mark all of the well-child checks in which they discuss a variety of health topics with patients. Analyzing the barriers perceived by PCPs as most important to preventing and treating obesity revealed trends regarding fast food consumption and physical inactivity. As shown in Table 2, even by their own report, most PCPs did not discuss fast foods at or prior to the twelve-month visit. At the eighteen-month visit fast foods are discussed by 32% of PCPs. Meanwhile, the two-year visit is the first well-child visit at which the majority of PCPs (68%) discuss fast food. Additionally, 5% of PCPs never discuss fast food at all with families. While the majority of PCPs ultimately discuss this topic, the discussion is not undertaken by a majority of PCPs at a specific encounter until the two- through five-year visits.

Fruit and vegetable discussion increases in frequency as the child ages, with a peak of 63% of PCPs discussing this at the twelve-month visit, before dropping to 51% at the eighteen-month visit. Between 22% and 26% of PCPs discuss having 3 meals per day by the twelve-month visit, whereas 65% are discussing meal frequency at the two- through five-year visits. Physical inactivity/exercise was another area of concern with 93% of PCPs recognizing this as a contribution to obesity, yet this topic was discussed by at most 23% of PCPs at and/or before the twelve-month visit. By the eighteen-month visit, at most 48% of PCPs had ever discussed the topic with their patients or families. The two- through five-year visits are the first time at which the majority of PCPs (68%) discuss this topic. The percent of well-child visits where screen time is discussed closely mirrors physical activity/exercise, and a similar trend is seen regarding the discussion of fast foods (Table 2).

## 4. Discussion and Conclusions

A major finding of the current study involved the relative importance PCPs ascribe to perceived barriers in treating obesity. Our results reproduced those of a previous study utilizing a similar survey but performed within a pediatric setting [11]. Our results confirm that family medicine PCPs share the same top six concerns when dealing with perceptions surrounding obesity. These concerns center around physical inactivity, fast food consumption, and motivation to change. The barriers identified were in areas that were inconsistently addressed in practice, specifically prior to the two-year well-child check. Despite the apparent lack of congruence between the identified barriers and the actions by PCPs in clinical practice, this study is limited in being a self-reported survey. Furthermore, due to the self-reported nature, PCPs may overestimate how often they address certain issues.

A second major finding of the current study was the inconsistency PCPs demonstrated concerning when discussion took place for physical activity, fruit and vegetable selection, screen time, juice, and other beverage choices. This study suggests that primary prevention interventions targeting obesity in practice are either misplaced or missed altogether in some cases, which is consistent with other recent studies demonstrating missed opportunity for primary prevention of obesity [21–24]. As this study is representative of an academic practice population with close geographic proximity, further study on a larger scale and in other practice populations would be warranted.

The AAP recommendations make no mention of fruits and vegetables until the five- and six-year well-child visits [6]. In another case, the AAP guidelines suggest the discussion of having 3 meals per day at the nine- and twelve-month well-child checks. However, in this study, only 26% of PCPs have this discussion specifically at or before the twelve-month well-child visit. Interestingly 65% of PCPs have this discussion at the two- through five-year well-child visits despite lack of specific recommendation to do so. PCPs seem to be aware of existing guidelines but, in this example, delay the delivery.

Other than this specific recommendation at the five-year well-child visit, all of the earlier well-child visit nutrition and diet guidelines are relatively nonspecific regarding diet composition. This likely contributes toward the variability observed in this study, as many PCPs chose to have discussions, such as fruits and vegetables, at differing well-child visits. Therefore, we believe that it would be advantageous to the goals of obesity prevention and treatment to have early and targeted interventions that precede adoption of adverse lifestyle choices [25–27]. Although it is beyond the scope of this paper, innovative approaches in obesity treatment have been identified over the past decade [28–30].

Comparing this study of family medicine PCPs with a similar study done with pediatric providers revealed some similarities in practice behavior. PCPs behavior in both studies reflected the general AAP anticipatory guidance guidelines pertaining to obesity [7, 11]. However, the anticipatory guidance discussions were not consistently performed at specific visits in either study, with greater variability observed in this family practice study (see Table 2). Different methodologies were used in data collection, as the study for family medicine PCPs was measured using a cumulative approach, whereas the pediatric study was measured at point of first intervention. In comparing these studies, we chose to use the most conservative estimates by summating the percentages of anticipatory guidance being discussed. For example, a single PCP may have indicated that they discussed fast food at both the four- and six-month visits. Our study would count both of these as unique interventions being done by different PCPs. Consequently, these findings may offer a realistic representation or, otherwise, an overestimation of how often topics were discussed at well-child checks.

The present study identifies discrepancies in PCPs adherence to counseling guidelines for nutrition, exercise, and screen time. In regard to exercise and screen time, this study demonstrates similar increases in the percentage of PCPs discussing these topics, with the majority doing so at the two- through five-year well-child checks, and a peak between the six- through eleven-year well-child checks. However, the AAP guidelines suggest that a majority of PCPs address each of these topics much earlier. It would be beyond the scope of this discussion to address all elements of the survey. Therefore, the remainder of this discussion will focus on the results relevant to fast food counseling and nutrition and their role in obesity prevention.

A surprising result from this study was that there is no clear consensus as to when PCPs are having discussions about fast food. While a prior study indicated that 62% of pediatricians addressed fast foods at or prior to twelve-month well-child visits, this study shows that at most 39% of family physicians did so (Table 2) [11]. These estimates suggest that there is still a large percentage of the population receiving no counseling on fast food during the entire first year of life. It is not until the two-year well-child visit that a majority of PCPs discuss fast food. Meanwhile, previous research has shown that there is a profound shift in dietary habits toward fast foods, such as French fries, that occurs between the one- and two-year well-child checks [8].

To address fast food consumption, PCPs could consider integrating a universal “French Fry Discussion” regularly at the twelve-month well-child care visit. Given the recognition that food transitioning towards fast foods such as French fries occurs within the subsequent window from twelve months to twenty-four months [8], it might be beneficial to offer a specific intervention at this visit. Furthermore, prior research has shown that a dedicated intervention targeting two-year olds and their families can significantly reduce BMI [31].

The “French Fry Discussion” could take the form of a purposeful talk with family members about the importance of avoiding fast foods, fried foods, and sweetened beverages. For example, using motivational interviewing techniques has been shown to be effective in changing behavior relating to obesity, and this approach may address such a complex behavior [32, 33]. This visit could additionally include handouts or printouts about alternative foods and snacks that are affordable and can be prepared quickly. This discussion can move beyond the unidirectional lecturing that often occurs. Rather, this should be an individualized discussion, in which family members are encouraged to voice their concerns and devise solutions that meet their unique situation. As such, we would not anticipate a discussion of this relevance requiring anything less than ten minutes of dedicated visit time. The goal of this discussion would be to leverage the patient-physician-family relationship to positively impact lifestyle choices for both the child and the family. Further study is needed to examine actual obesity prevention and treatment guidelines in clinical practice and to discern the specifics, feasibility, and benefits of incorporating additional counseling into routine well-child care.

## Figures and Tables

**Table 1:** Respondents' perceptions of barriers to obesity prevention or treatment in primary care, expressed as the percentage of respondents providing affirmative responses to the statement: “Please rate the importance of each of the following general barriers to preventing or treating overweight or obesity in children in your professional experience” (on a scale of importance 1–5).

	3	4	5	(4 + 5)
	Moderately Important	Very Important	Critically Important	Total Very and Critically Important
Parent is unaware that child is overweight	25%	47%	16%	63%
Parent is not motivated to change diet or lifestyle∗	18%	40%	40%	81%
Child is not motivated to change diet or lifestyle∗	16%	45%	30%	75%
Parents are overweight, so they are not concerned that child is overweight	26%	44%	28%	72%
Overweight child does not act sick	32%	32%	2%	33%
Overweight child is a “good eater”∗	39%	27%	4%	30%
Parents do not have time to shop for healthier foods	28%	35%	18%	53%
Families are too busy to eat home cooked meals	21%	46%	25%	70%
Families often have fast food meals∗	11%	35%	51%	86%
Families are too busy to eat meals together	30%	41%	14%	55%
Families watch too much TV∗	12%	51%	28%	79%
Families do not get enough exercise∗	4%	21%	71%	93%
Healthy foods are too expensive	27%	36%	34%	70%
Healthy lifestyle habits are too complicated to follow	46%	32%	5%	38%
Published reports of research studies about diet and nutrition are often confusing	35%	26%	4%	30%
TV advertisements promote unhealthy foods	23%	37%	25%	61%
School lunches promote unhealthy eating habits	39%	33%	19%	53%
PCPs have limited time to discuss nutrition	29%	36%	29%	64%
PCPs are frustrated with the low success rate of treating overweight children	25%	42%	19%	61%
PCPs lack sufficient training to help overweight children	36%	34%	11%	45%
PCPs lack sufficient education materials to prevent or treat overweight children	28%	49%	7%	56%
PCPs are not adequately compensated for treating obesity	39%	23%	16%	39%
It is difficult to convince parents to see a nutritionist	28%	42%	12%	54%
There are not enough nutritionists to help with overweight children	23%	36%	20%	55%
The PCPs weight status or body mass index	32%	13%	2%	14%

^*^The top 5 barriers that respondents rated as either Very Important or Critically Important.

**Table 2:** Distribution of well-child checks at which respondents address obesity-related topics during childhood and adolescence expressed as the percentage providing affirmative responses to the question “At which well-child check(s) do you typically talk about the following? Mark all that apply.”

	Newborn	2 months	4 months	6 months	12 months	18 months	2–5 years	6–11 years	12–18 years	None
Cereals	13%	20%	54%	57%	43%	25%	18%	14%	11%	2%
Juice	12%	17%	37%	62%	67%	50%	42%	31%	27%	6%
Non-juice sugar sweetened beverages	12%	10%	15%	33%	54%	44%	48%	46%	46%	6%
Fruits and vegetables	4%	7%	21%	58%	63%	51%	54%	51%	47%	2%
Sippy cups	2%	6%	15%	44%	74%	37%	17%	0%	0%	0%
Finger foods	0%	2%	11%	51%	67%	31%	13%	4%	5%	5%
Fast foods	0%	0%	4%	9%	27%	32%	68%	68%	59%	5%
Candy	0%	2%	9%	9%	34%	42%	62%	55%	43%	15%
Screen time (TV, video, video games, computer, texting)	0%	2%	4%	11%	25%	30%	71%	73%	66%	4%
Physical activity/exercise	0%	0%	0%	2%	21%	25%	68%	79%	70%	2%
Sleep	42%	39%	37%	35%	40%	39%	47%	47%	56%	5%
Having 3 meals/day (not skipping breakfast, lunch, or dinner)	0%	0%	0%	4%	22%	17%	65%	59%	63%	15%
